# Growth Hormone Is Beneficial for Induction of Spermatogenesis in Adult Patients With Congenital Combined Pituitary Hormone Deficiency

**DOI:** 10.3389/fendo.2022.868047

**Published:** 2022-04-22

**Authors:** Yiyi Zhu, Min Nie, Xi Wang, Qibin Huang, Bingqing Yu, Rui Zhang, Junyi Zhang, Bang Sun, Jiangfeng Mao, Xueyan Wu

**Affiliations:** National Health Commission (NHC) Key Laboratory of Endocrinology, Department of Endocrinology, Peking Union Medical College Hospital, Peking Union Medical College, Chinese Academy of Medical Sciences, Beijing, China

**Keywords:** congenital combined pituitary hormone deficiency, recombinant human growth hormone, gonadotropin, spermatogenesis, insulin-like growth factor 1

## Abstract

**Background:**

Gonadotropins are effective in inducing spermatogenesis in patients with congenital combined pituitary hormone deficiency (CCPHD). Data on recombinant human growth hormone(rhGH) adjuvant treatment to improve gonadotropin-induced spermatogenesis are limited.

**Design and Setting:**

This retrospective study included 60 male patients with CCPHD on a relatively large case series in a single center from mainland China. Twenty-nine patients who received gonadotropin therapy alone were defined as the Gn group, while 31 patients treated with a combination of rhGH and gonadotropins were defined as GH/Gn group.

**Results:**

Spermatogenesis rate was 96.77% (30/31) and 62.07% (18/29) in the GH/Gn and Gn group, respectively (P < 0.001). The time for initial sperm appearance in the GH/Gn group was shorter than in the Gn group (14 versus 23 months, P < 0.001). A higher level of serum testosterone was achieved in the GH/Gn group than in the Gn group (4.79 versus 3.38 ng/mL, P = 0.026). After adjustment for potential confounders, rhGH supplementation was an independent beneficial factor on spermatogenesis (HR = 2.294, 95% CI: 1.143-4.604, P = 0.019).

**Conclusions:**

rhGH induces earlier spermatogenesis in patients with CCPHD, which encourages the co-treatment with rhGH and gonadotropins in CCPHD patients.

## Introduction

Congenital combined pituitary hormone deficiency (CCPHD) is a disease characterized by pituitary hypoplasia and multiple anterior pituitary hormone deficiencies. In association with an interrupted pituitary stalk and/or ectopic posterior pituitary, these patients are usually known as pituitary stalk interruption syndrome (PSIS) (1). Growth hormone deficiency (GHD) happens in nearly 100% of patients with CCPDH, and hypogonadotropic hypogonadism (HH) occurs in 96% of patients (2). Therefore, these patients dominantly present with growth retardation, pubertal delay, as well as infertility in adults. Under this circumstance, sex hormone replacement is regarded as the primary therapy to develop secondary sexual characteristics. When fertility is required, gonadotropins therapy is effective in the induction of spermatogenesis (3).

Recombinant human growth hormone (rhGH) replacement promotes linear growth before epiphyseal closure. After entering adulthood, rhGH replacement mainly improves lipoprotein metabolism, body composition, and bone mineral density (4). Few studies focus on the efficacy of rhGH therapy in spermatogenesis (5–7). No consensus is reached on rhGH adjuvant treatment to improve spermatogenesis. And most of these studies discuss rhGH treatment in gametogenesis of non-GHD adults. Such data on GHD males are scanty.

Indeed, the growth hormone/insulin-like growth factor 1 (GH/IGF-1) and the hypothalamic-pituitary-gonadal (HPG) axes are closely interconnected (8). Especially in female reproduction, GH/IGF-1 regulates follicular development and oocyte maturation in a gonadotropin-dependent status (9). In males, most evidence comes from animal experiments. In IGF-1 gene null, –/– mutant mice (10), the testes, epididymides duct, vas deferens, seminal vesicles, and prostate are vestigial, leading to a low spermatogenesis rate at 18% of the normal level. Also, the male mutants are presented with a reduced level of serum testosterone, which is correlated with the developmental delay of Leydig cells. In the GH deficient Snell dwarf mice, seminiferous tubules are underdeveloped and germ cells are reduced, resulting in infertility (11). These results indicate that reduced GH/IGF-1 secretion impairs HPG function. The induction of IGF-1 secretion by GH treatment can partially recover the impaired HPG axes (12). Therefore, in this study, we aim to evaluate sperm production under rhGH adjuvant therapy with gonadotropins in CCPHD male patients.

## Materials and Methods

### Diagnosis of Congenital Combined Pituitary Hormone Deficiency

Combined pituitary hormone deficiency (CPHD) is defined as at least two anterior pituitary hormone deficiencies (13). Radiological presentations include pituitary gland hypoplasia, broken or slim pituitary stalk, and ectopic neurohypophysis (1).

Growth hormone deficiency (GHD) is defined as a peak GH level below 10 µg/L after two GH provoking tests (insulin-induced hypoglycemic GH stimulating test and L-dopa GH stimulating test) or biochemical evidence of multiple pituitary hormone deficiencies (≥3 pituitary hormone deficiencies) together with low-serum IGF-1 levels (< –2.0 SDS) (14). Hypogonadotropic hypogonadism (HH) is defined as low serum levels of testosterone (< 3.47 nmol/L) with low or normal serum levels of gonadotropins. Central hypothyroidism refers to free thyroxin (FT4) below 1.0 ng/dL in conjunction with a low or normal thyroid-stimulating hormone (TSH). Central adrenal insufficiency refers to morning cortisol levels below 5 μg/dL without elevated adrenocorticotrophic hormone (ACTH).

### Patients

The study was approved by the ethics committee of Peking Union Medical College Hospital (PUMCH). The medical record of each patient was collected. Patients were followed up in PUMCH, from December 2019 to December 2021.

Clinical presentations, cryptorchidism, and delivery status were collected from the medical records. Sellar MRI was assessed by sagittal and coronal T1-weighted sequences and axial T2-weighted sequences. Testicular volume was measured (by a Prader orchidometer) and biochemical indexes were documented at each visit. All the clinical information and imaging examinations were reviewed by a board-certified endocrinologist and radiologist, respectively.

**Figure 1:**
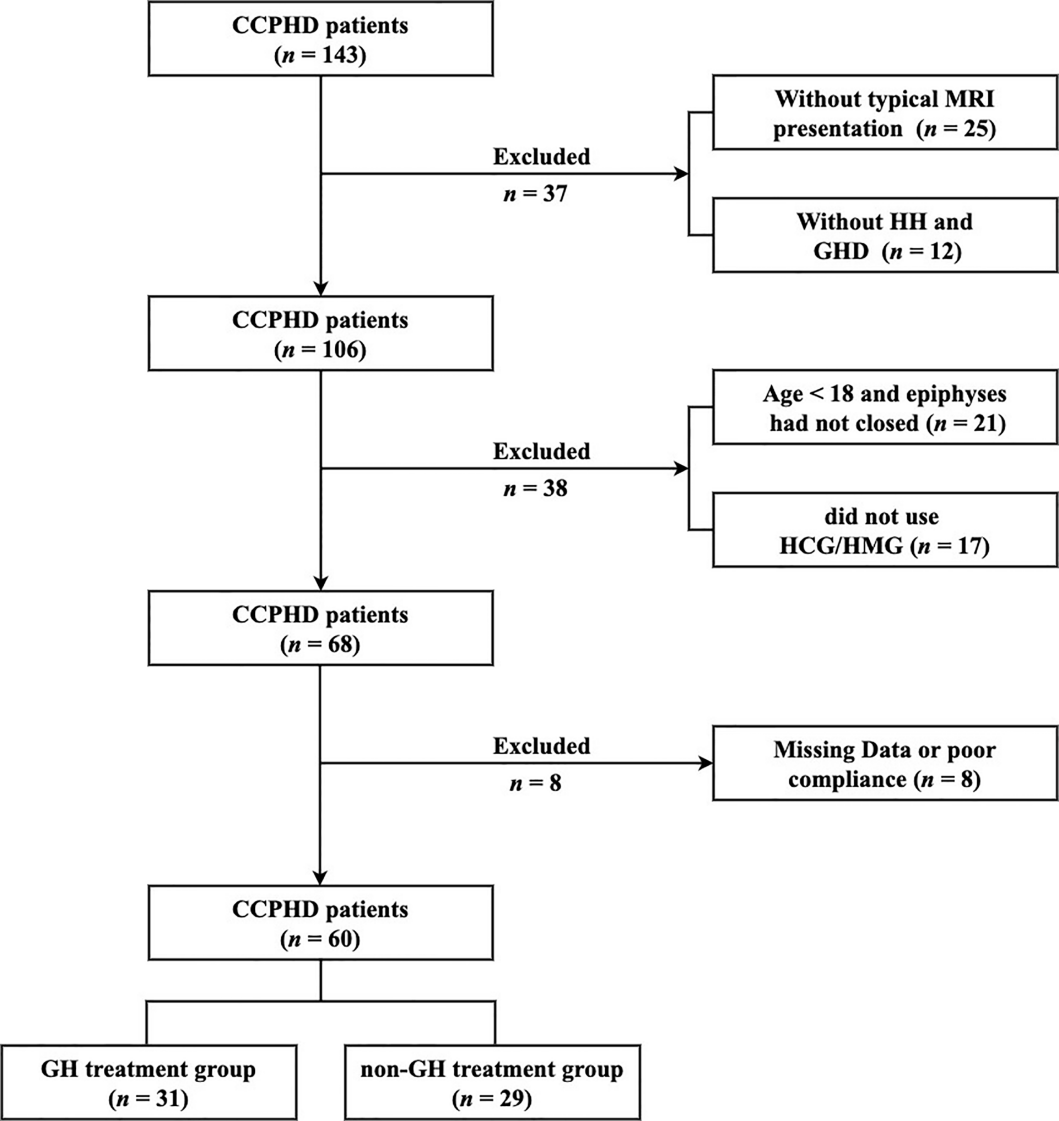
The flowchart of patient screening. CCPHD, Congenital combined pituitary hormone deficiency; MRI, Magnetic resonance imaging; HH, Hypogonadotropic hypogonadism; GHD, Growth hormone deficiency; HCG, Human chorionic gonadotrophin; HMG, Human menopausal gonadotrophin.

Patients were selected based on the following criteria: (i) typical MRI presentations of PSIS; (ii) combined anterior pituitary hormone deficiencies, at least including GHD and HH; (iii) age > 18 years; (iv) male with a desire for fertility. Exclusion criteria were as follows: (i) acquired hypopituitarism, including tumor, surgery, injury, and infection in hypothalamus areas; (iii) lack of follow-up information; Finally, 60 CCPHD patients were included in this study (**Figure 1**).

### Treatment and Follow-up

Once the diagnosis of hypopituitarism was made, hormone replacement therapy was recommended (15). Physiologic dosage of glucocorticoids (hydrocortisone 10-20 mg daily) was administered when adrenal insufficiency was diagnosed. L-Thyroxin was adopted to achieve serum FT4 levels within the normal range. For fertility (16), human chorionic gonadotropin (HCG) 5000 U (Livzon Pharmaceutical Co, Guangdong, China) and human menopausal gonadotropin (HMG) 150 U (Livzon Pharmaceutical Co, Guangdong, China) were intramuscularly injected once a week, which was keeping the same dosage during the follow-up. rhGH replacement therapy (GeneScience Pharmaceuticals Co, Changchun, China) was used in 31 patients. And other 29 patients refused rhGH therapy. The dose of rhGH was started with 0.2 mg/d and was titrated to maintain IGF-1 levels within the age-adjusted normal range (17). All patients were divided into GH/Gn group (n = 31) and the Gn group (n = 29) according to using rhGH replacement or not. Participants were followed at an interval of 3 to 6 months. The main outcome was spermatogenesis (≥1 sperm/ejaculate). The secondary outcome included time of first sperm appearance, semen parameters, testicular volume, and serum testosterone level. All the indicators were measured on each visit. The serum levels of testosterone were determined 48 hours after HCG/HMG injection.

### Hormone Assays and Seminal Analysis

Venous blood samples were taken in a fasting state at 8:00 AM. Luteinizing hormone (LH), follicle-stimulating hormone (FSH), and total testosterone were measured using commercial kits by chemiluminescence (ACS:180 Automatic Chemiluminescence Systems, Bayer, Germany). GH and IGF-1 were determined by chemiluminescent enzyme immunometric assay (Immulite 2000, Siemens Healthcare Diagnostics, UK). IGF-1 was transformed to SDS values according to the reference data reported by Bidlingmaier (17). Other hormonal indicators including free triiodothyronine (FT3), FT4, TSH, and cortisol (at 8:00 am), were routinely measured with standardized methods at the Department of Clinical Laboratory at PUMCH.

Semen samples were collected by masturbation after sexual abstinence for 3-5 days. Data on sperm concentration and motility were analyzed according to the standard procedures provided by the World Health Organization method (18). Successful spermatogenesis was defined as more than one spermatozoon appearing in the unprocessed or centrifuged seminal fluid.

### Statistical Analysis

SPSS version 25.0 was used for data analysis. Normally distributed data were expressed as Mean ± SD, while non-normal distribution data were expressed as median (25^th^ percentile, 75^th^ percentile). Differences between the two groups were analyzed by Student t-test or nonparametric Wilcoxon-Mann-Whitney test. Qualitative data were described as percentages and analyzed using the Chi-square test or Fisher’s exact test. Kaplan-Meier and Mantel-Cox (log-rank test) analyses were applied to estimate the median spermatogenic time to achieve a threshold sperm level. Multivariable Cox regression analysis assisted in adjusting for confounders to analyze the effect of growth hormone therapy on spermatogenesis. The age of initial treatment, body mass index (BMI), basal LH level, basal FSH level, basal testicular size, serum testosterone level after treatment, and cryptorchid (no/yes) were subjected to Cox regression as adjustable variables. The *P*-value reported was two-sided and a value of less than 0.05 was considered statistically significant.

## Results

### Baseline Clinical Features of CCPHD Patients

In 60 male CCPHD patients who were retrospectively evaluated, the age for starting treatment was 27.09 ± 5.46 years. Most of them had malpresentation (57/60, 95%), in either breech or foot presentation. Small testis was commonly seen with a median testicular size of 3 mL. Cryptorchidism was less frequent (2/60, 3%). The prevalence of hormone deficiency was 100% (60/60) in GH, 100% (60/60) in LH/FSH, 85% (51/60) in TSH, and 73% (44/60) in ACTH. Based on personal preference for rhGH treatment or not, patients were divided into GH/Gn or Gn groups. No statistical difference was observed between the two groups in terms of anthropometry parameters and basal hormone concentrations (*P* > 0.05). Specifically, basal IGF-1 SDS was -6.43 ± 1.29 versus -6.74 ± 1.34 (*P* = 0.287) in the GH/Gn and Gn group; basal LH level was 0.20 (0.05, 0.55) versus 0.20 (0.00, 0.31) IU/L (*P* = 0.220); and basal testosterone level was 0.10 (0.05, 0.28) versus 0.10 (0.03, 0.10) ng/mL (*P* = 0.205), respectively. The basal clinical manifestations were summarized in **Table 1**.

**Table 1:** The baseline features of male patients with CCPHD.

Parameter	Total (n = 60)	GH/Gn (n = 31)	Gn (n = 29)	Reference range	*P*-value
Anthropometry parameters before therapy					
Age at treatment (years)	27.09 ± 5.46	27.64 ± 5.29	26.50 ± 5.66	—	0.421
Malpresentation, n (%)	57 (95.00)	28 (90.32)	27 (93.10)	—	0.679
Final Height (cm)	172.32 ± 8.88	172.00 ± 10.13	172.66 ± 7.50	—	0.778
BMI (kg/cm^2^)	24.19 ± 4.22	23.66 ± 4.34	24.77 ± 4.08	—	0.360
Basal testicular size (mL)	3.00 (2.00, 4.00)	2.00 (2.00, 4.00)	3.00 (2.00, 4.50)	24.7 ± 1.6	0.613
Cryptorchidism, n (%)	2 (3.33)	1 (3.22)	1 (3.45)	—	0.962
Basal hormone concentrations					
IGF-1 (ng/mL)	25.00 (25.00, 39.75)	25.00 (25.00, 41.00)	25.00 (25.00, 33.50)	—	0.809
IGF1 SDS	-6.58 ± 1.15	-6.43 ± 1.29	-6.74 ± 1.34	—	0.287
LH (IU/L)	0.20 (0.00, 0.49)	0.20 (0.05, 0.55)	0.20 (0.00, 0.31)	1.24-8.62	0.220
FSH (IU/L)	0.66 (0.23, 1.69)	0.57 (0.20, 1.80)	0.70 (0.30, 1.46)	1.27-19.26	0.912
Testosterone (ng/mL)	0.10 (0.05, 0.26)	0.10 (0.05, 0.28)	0.10 (0.03, 0.10)	1.75-7.81	0.205
TSH (uIU/L)	2.03 (0.35, 3.35)	1.97 (0.23, 2.79)	2.22 (0.60, 3.61)	0.38-4.34	0.240
Free thyroxin (ng/dL)	0.75 (0.59, 0.96)	0.75 (0.68, 0.94)	0.72 (0.55, 1.02)	0.18-1.89	0.379
Cortisol (8 am) (μg/dL)	4.46 (1.87, 8.97)	5.80 (2.30, 9.74)	3.08 (1.80, 6.98)	4.00-22.30	0.153
Pituitary hormone deficiency					
GH deficiency, n (%)	61 (100)	32 (100)	29 (100)	—	–
LH/FSH deficiency, n (%)	61 (100)	32 (100)	29 (100)	—	–
TSH deficiency, n (%)	51 (85.00)	26 (83.87)	25 (86.20)	—	0.800
ACTH deficiency, n (%)	44 (73.33)	21 (67.74)	23 (79.31)	—	0.311

P < 0.05 was considered statistically significant.

CCPHD, Congenital combined pituitary hormone deficiency; BMI, Body mass index; IGF-1, Insulin-like growth factor 1; LH, Luteinizing hormone; FSH, Follicle-stimulating hormone; TSH, Thyroid-stimulating hormone; GH, Growth hormone; ACTH, Adrenocorticotrophic hormone.

### Hormonal Levels After Therapy in Two Groups

The treatment duration was 21.50 (13.25, 27.50) months for all participants, and no difference between the two groups was observed (*P* = 0.711) (**Table 2**). During hormonal replacement therapy, IGF-1 SDS increased obviously in the GH/Gn group than the Gn group (-0.40 vs. -4.60, *P* < 0.001). Following the gonadotropin therapy with the same dose and administration frequency, a higher level of serum testosterone level was achieved in the GH/Gn treatment group than the Gn group (4.79 vs. 3.38 ng/mL, *P* = 0.026). There were 21 patients in the GH/Gn group receiving hydrocortisone replacement, while 23 patients in the Gn group taking hydrocortisone. Dosage of hydrocortisone replacement had no statistical difference between the two groups, with a daily average of 10.00 mg in both groups (*P* = 0.467). A total of 26 patients received L-T4 treatment in the GH/Gn group and 25 patients in the Gn group. The average daily dosage of L-T4 treatment was 100.00(50.00, 100.00) μg and 75.00 (50.00, 100.00) μg in GH/Gn and Gn groups, respectively (*P* = 0.195). After stable replacement, FT4 levels remained similar between the two groups (*P* = 0.965).

**Table 2:** Hormone levels and semen quality after therapy. .

Parameter	Total (n = 60)	GH/Gn (n = 31)	Gn (n = 29)	Reference range	*P*-value
Treatment duration (months)	21.50 (13.25, 27.50)	22.00 (11.50, 30.00)	21.00 (17.00, 25.00)^a^	—	0.711
Daily dosage of hydrocortisone (mg/day)	10.00 (1.25, 15.00)	10.00 (0.00, 15.00)	10.00 (5.00, 15.00)	—	0.467
Daily dosage of LT-4 (μg/day)	75.00 (50.00, 100.00)	100.00 (50.00, 100.00)	75.00 (50.00, 100.00)		0.195
IGF-1 (ng/mL)	143.50 (62.50, 223.50)	222.00 (176.00, 262.00)	61.00 (45.50, 90.50)	—	<0.001^***^
IGF-1 SDS	-2.04 (-4.55, -0.40)	-0.40 (-1.10, 0.90)	-4.60 (-5.80, -3.55)	—	<0.001^***^
Free thyroxin (ng/dL)	1.12 ± 0.06	1.12 ± 0.05	1.12 ± 0.09	0.18-1.89	0.965
Testosterone (ng/mL)	3.99 (2.51, 5.37)	4.79 (2.77, 6.10)	3.38 (2.31, 4.59)	1.75-7.81	0.026^*^
Testicular size (mL)	8.00 (4.25, 12.00)	8.00 (5.00, 12.00)	6.00 (4.00, 12.00)	—	0.143
Spermatogenesis (≥1 sperm/ejaculate),n (%)	48 (80.00)	30 (96.77)	18 (62.07)	—	<0.001^***^
Time of first sperm appearance (months)	17.00(12.00, 24.00)	14.00(10.00, 19.00)	23.00(15.00, 33.00)	—	<0.001^***^
Sperm concentration [10^6^/ml; median (quartiles)]	8.02 (0.00, 19.74)	6.09 (0.00, 18.81)	10.52 (1.59, 24.91)	17.0-83.5	0.292
Sperm progressive motility (A + B) (%)	0.24 (0.00, 0.49)	0.27 (0.00, 0.50)	0.23 (0.04, 0.38)	—	0.813
Sperm total mobility (A + B + C) (%)	0.27 (0.00, 0.56)	0.30 (0.00, 0.56)	0.26 (0.04, 0.47)	—	0.948

P < 0.05 was considered statistically significant, *P < 0.05, ***P < 0.001.

CCPHD, Congenital combined pituitary hormone deficiency; IGF-1, Insulin-like growth factor 1.

a The treatment duration only included the usage of hydrocortisone, LT-4, and gonadotropin.

### Spermatogenesis After Therapy in Two Groups

**Figure 2:**
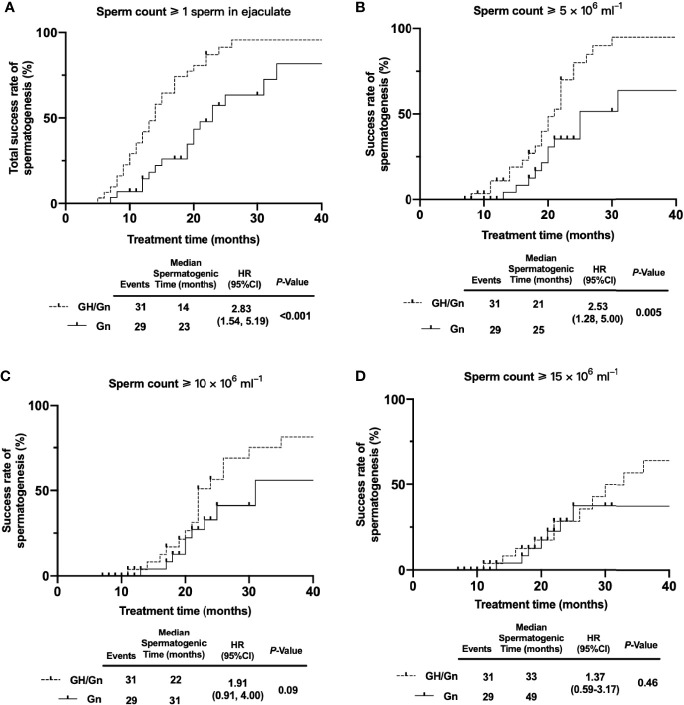
A time-to-event analysis when sperm concentration ≥ 1, ≥ 5, ≥ 10, and ≥ 15 million/ml. **(A)** The median time to begin sperm production (≥1 sperm) in GH/Gn group is shorter than that in the Gn treatment group (*P < 0.001*). **(B)** The median time of reaching a sperm threshold ≥ 5 × 10^6^ ml^−1^ in GH/Gn group is shorter than that in the Gn treatment group (*P = 0.005*). **(C)** The median time of reaching a sperm threshold ≥ 10 × 10^6^ ml^−1^ is no statistically significant between the two groups (*P = 0.09*). **(D)** The median time of reaching a sperm threshold ≥ 15 × 10^6^ ml^−1^ is no statistically significant between the two groups (*P = 0.46*).

At the initial diagnosis, patients had a poor reproductive system development and were presented with small testis and penis, owing to low basal LH/FSH and testosterone levels. The erectile dysfunction made male patients unable to collect seminal fluid for semen analysis. Based on the same gonadotropin therapy, patients who received rhGH adjuvant therapy had a higher spermatogenesis rate (*P* < 0.001) and a shorter time of first sperm appearance (*P* < 0.001), compared to the Gn group. Specifically, the success rates of gametogenesis were 96.77% (30/31) and 62.07% (18/29) in rhGH and non-rhGH treatment groups, respectively. Meanwhile, a time-to-event (spermatogenesis) analysis was conducted in two groups. The median time of sperm appearance in semen in the GH/Gn and Gn groups was 14 months and 23 months, respectively (*P* < 0.001, **Figure 2A**). Duration for reaching a sperm concentration threshold ≥ 5 × 10^6^ ml^−1^ was 21 months vs. 25 months in the GH/Gn and Gn groups (*P* = 0.005, **Figure 2B**). The time required for reaching a sperm concentration threshold ≥ 10× 10^6^ ml^−1^ and 15× 10^6^ ml^−1^, however, was not significantly different between these two groups. (**Figures 2C, D** **)**. At the final visit, sperm concentration remained similar between two groups (6.09 (0.00, 18.81) ml^−1^ *vs*. 10.52 (1.59, 24.91) ml^−1^, *P* = 0.292). The sperm progressive motility (defined by WHO grades a + b) and sperm total mobility (defined by WHO grades a + b + c) were similar between the two groups. The GH/Gn group had a trend of having a larger testicular size than the Gn group (8 mL *vs*. 6 mL, *P* = 0.143).

### Predictive Factors for Spermatogenesis in CCPHD Patients

In the univariate analysis using the Cox model, rhGH adjuvant treatment was a favorable predictor for the shortest time to spermatogenesis (HR = 2.832, 95% CI: 1.543-5.198, *P* < 0.001). While after controlling for age of starting treatment, BMI, basal LH level, basal FSH level, basal testicular size, and cryptorchid, rhGH treatment (HR = 2.294, 95% CI: 1.143-4.604, *P* = 0.019) was still a positive predictor for the shortest time to spermatogenesis (**Table 3**).

**Table 3:** Hazard ratios (95% CIs) of rhGH replacement to spermatogenesis.

Variable	Univariable model		Multivariable model	
	Hazard Ratio (95% CI)	*P-*Value	Hazard Ratio (95% CI)	*P*-Value
Age at treatment (years)	1.008 (0.956, 1.062)	0.781	1.004 (0.948, 1.064)	0.890
BMI (kg/cm^2^)	0.962 (0.894, 1.035)	0.298	0.982 (0.897, 1.074)	0.686
Basal LH (IU/L)	1.819 (1.062, 3.116)	0.029^*^	2.163 (0.949, 4.930)	0.066
Basal FSH (IU/L)	1.094 (0.826, 1.449)	0.529	0.974 (0.640, 1.482)	0.902
Basal testicular size (mL)	1.258 (1.078, 1.467)	0.003^**^	1.223 (1.017,1.469)	0.032^*^
rhGH replacement (no/yes)^a^	2.832 (1.543, 5.198)	<0.001^***^	2.294 (1.143, 4.604)	0.019^*^
Testosterone after treatment (ng/mL)	1.092 (0.964, 1.238)	0.168	1.006 (0.867, 1.167)	0.939
Cryptorchid (no/yes)^b^	1.330 (0.320, 5.538)	0.695	1.940 (0.432, 8.706)	0.387

P < 0.05 was considered statistically significant, *P < 0.05, **P < 0.01, ***P < 0.001.

BMI, Body mass index; LH, Luteinizing hormone; FSH, Follicle-stimulating hormone; rhGH, Recombinant human growth hormone.

a use non-rhGH replacement as contrast.

b use no cryptorchid as contrast.

## Discussion

In our study, a higher success rate of spermatogenesis and shorter treatment duration for first sperm appearance was observed in CCPHD patients receiving adjuvant rhGH therapy, compared to receiving gonadotropins alone. For the first time, it provided direct evidence that normalization of GH/IGF1 could improve the gametogenesis in CCPHD patients.

Previous studies showed inconsistent results on the effects of rhGH therapy to induce spermatogenesis. Most of the researches did not encourage the additional use of rhGH to promote spermatogenesis because no improvement of sperm quantity and quality was seen neither in the non-GHD patients (5, 6) nor the acquired GHD patients (7). These conclusions did not conform to our results due to the different populations selected. CCPHD is a good model to explore the interaction between the somatotropic and testicular axis and is accompanied by complete hormonal deficiency at birth. The replacement of rhGH and gonadotropins mimics a pubertal developmental course and secures the resumption of growth and development of the “dormant” sexual organs, which gives intuitive evidence of the crosstalk between GH/IGF-1 and gonadotropins in sexual maturation and development. Therefore, our conclusion is more convincing and rhGH supplementation is recommended for gonadotropin-induced spermatogenesis in CCPHD patients.

In our study, CCPHD patients with rhGH treatment showed a tendency for a shorter time of spermatogenesis, although successful spermatogenesis could also happen in 62.07% of CCPHD patients receiving gonadotropins therapy alone. This phenomenon is in line with the clinical evidence for the role of GH/IGF-1 in the maturation of the reproductive system. In GH insensitivity patients, also known as Laron syndrome, the onset of puberty delays to the age of 15.6 ± 2.6 years and the duration of the pubertal development procession elongates to 6.2 ± 0.6 years (19). Despite delayed puberty being common in Laron syndrome, full maturity of the reproductive system is reached spontaneously in the end (8, 19). The possible reason might be the local production of IGF-1 in testis by the way of autocrine or paracrine, under the control of FSH and LH (20). In a conclusion, although rhGH is not indispensable for sperm production, it assists in accelerating the progression of pubertal development and speeding up sperm appearance in a relatively shorter period.

Several factors may influence spermatogenesis, including the age of initial treatment, BMI (21), basal LH level (22), basal FSH level (22, 23), basal testicular size (24), and cryptorchid history (24). After adjusting for confounding factors, rhGH treatment was associated with a 129% higher success rate of spermatogenesis, demonstrating rhGH had a beneficial potential for sperm induction.

Furthermore, higher serum levels of testosterone in the GH/Gn group, a reliable biomarker of a better response of Leydig cell to HCG, may be another contributor for earlier sperm production. Previous studies demonstrated that rhGH therapy increased HCG-induced testosterone secretion in hypopituitary boys (25, 26) and adults (7). We found that serum testosterone levels in GH/Gn group had a higher level of 4.79 ng/mL compared to the Gn group, consistent with previous studies. Therefore, our study added to evidence that rhGH and gonadotropins had some synergistic effects on Leydig cells for testosterone production.

The main advantages of our study were the relatively large sample size and long follow-up period. Supplementary thyroid hormone, glucocorticoids, and gonadotropins were high balanced between the two groups, which helped to exclude most of the interventional compound factors. On the other hand, there still existed gaps and limitations. First, pertinent limitations are familiar to all retrospective cohort studies, including recall bias and information bias. Thus, prospective, well-designed, randomized controlled trials are needed to validate the causative effects of rhGH on spermatogenesis. Second, the molecular mechanisms of the synergistic effect between GH/IGF1 and gonadotropins have not yet been fully explored because of the limitation of research conditions.

## Conclusion

Our study reveals that supplementary rhGH may promote earlier spermatogenesis and higher testosterone production in patients with CCPHD than gonadotropin alone. Practically, additional rhGH should be recommended to CCPHD patients for the induction of male fertility.

## Data Availability Statement

The raw data supporting the conclusions of this article will be made available by the authors, without undue reservation.

## Ethics Statement

The studies involving human participants were reviewed and approved by the ethics committee of Peking Union Medical College Hospital. Written informed consent for participation was not required for this study in accordance with the national legislation and the institutional requirements.

## Author Contributions

YZ: collecting the clinical data and writing the article. XW, MN, QH, and BY: patients follow-up and collecting clinical data. RZ, JZ, and BS: conducting the statistical analysis. JM and XYW: study design and polishing the article. All authors contributed to the article and approved the submitted version.

## Funding

The study was supported by the National Natural Science Foundation of China, No. 81771576 and No. 81971375; the Beijing Municipal Natural Science Foundation, No. 7202151 and No. 7212080.

## Conflict of Interest

The authors declare that the research was conducted in the absence of any commercial or financial relationships that could be construed as a potential conflict of interest.

## Publisher’s Note

All claims expressed in this article are solely those of the authors and do not necessarily represent those of their affiliated organizations, or those of the publisher, the editors and the reviewers. Any product that may be evaluated in this article, or claim that may be made by its manufacturer, is not guaranteed or endorsed by the publisher.

## References

[1] Vergier J Castinetti F Saveanu A Girard N Brue T Reynaud R. DIAGNOSIS OF ENDOCRINE DISEASE: Pituitary Stalk Interruption Syndrome: Etiology and Clinical Manifestations. Eur J Endocrinol (2019) 181(5):R199–209. doi: 10.1530/EJE-19-0168 31480013

[2] Guo Q Yang Y Mu Y Lu J Pan C Dou J. Pituitary Stalk Interruption Syndrome in Chinese People: Clinical Characteristic Analysis of 55 Cases. PloS One (2013) 8(1):e53579. doi: 10.1371/journal.pone.0053579 23341953 PMC3544917

[3] Mao J Xu H Wang X Huang B Liu Z Zhen J. Congenital Combined Pituitary Hormone Deficiency Patients Have Better Responses to Gonadotrophin-Induced Spermatogenesis Than Idiopathic Hypogonadotropic Hypogonadism Patients. Hum Reprod (2015) 30(9):2031–7. doi: 10.1093/humrep/dev158 26141714

[4] Gasco V Caputo M Lanfranco F Ghigo E Grottoli S. Management of GH Treatment in Adult GH Deficiency. Best Pract Res Clin Endocrinol Metab (2017) 31(1):13–24. doi: 10.1016/j.beem.2017.03.001 28477728

[5] Zalel Y Draysen E Goldschmit R Zadik Z Shoham Z. A Prospective Pilot Study of Co-Treatment With Growth Hormone and Gonadotropins for Improving Spermatogenesis in Normogonadotropic Patients With Severe Oligoteratoasthenospermia. Gynecol Endocrinol (1996) 10(1):23–8. doi: 10.3109/09513599609041266 8737188

[6] Giagulli VA. Absence of Effect of Recombinant Growth Hormone to Classic Gonadotropin Treatment on Spermatogenesis of Patients With Severe Hypogonadotropic Hypogonadism. Arch Androl (1999) 43(1):47–53. doi: 10.1080/014850199262724 10445104

[7] Carani C Granata AR De Rosa M Garau C Zarrilli S Paesano L. The Effect of Chronic Treatment With GH on Gonadal Function in Men With Isolated GH Deficiency. Eur J Endocrinol (1999) 140(3):224–30. doi: 10.1530/eje.0.1400224 10216517

[8] Chandrashekar V Zaczek D Bartke A. The Consequences of Altered Somatotropic System on Reproduction. Biol Reprod (2004) 71(1):17–27. doi: 10.1095/biolreprod.103.027060 15028633

[9] Dosouto C Calaf J Polo A Haahr T Humaidan P. Growth Hormone and Reproduction: Lessons Learned From Animal Models and Clinical Trials. Front Endocrinol (Lausanne) (2019) 10:404. doi: 10.3389/fendo.2019.00404 31297089 PMC6607366

[10] Baker J Hardy MP Zhou J Bondy C Lupu F Bellvé AR. Effects of an Igf1 Gene Null Mutation on Mouse Reproduction. Mol Endocrinol (1996) 10(9):903–18. doi: 10.1210/mend.10.7.8813730 8813730

[11] Matsushima M Kuroda K Shirai M Ando K Sugisaki T Noguchi T. Spermatogenesis in Snell Dwarf Little and Congenitally Hypothyroid Mice. Int J Androl (1986) 9(2):132–40. doi: 10.1111/j.1365-2605.1986.tb00876.x 3793256

[12] Chandrashekar V Bartke A. Induction of Endogenous Insulin-Like Growth Factor-I Secretion Alters the Hypothalamic-Pituitary-Testicular Function in Growth Hormone-Deficient Adult Dwarf Mice. Biol Reprod (1993) 48(3):544–51. doi: 10.1095/biolreprod48.3.544 8452930

[13] Castinetti F Reynaud R Quentien MH Jullien N Marquant E Rochette C. Combined Pituitary Hormone Deficiency: Current and Future Status. J Endocrinol Invest (2015) 38(1):1–12. doi: 10.1007/s40618-014-0141-2 25200994

[14] Yuen KCJ Biller BMK Radovick S Carmichael JD Jasim S Pantalone KM. American Association of Clinical Endocrinologists and American College of Endocrinology Guidelines for Management of Growth Hormone Deficiency in Adults and Patients Transitioning From Pediatric to Adult Care. Endocr Pract (2019) 25(11):1191–232. doi: 10.4158/GL-2019-0405 31760824

[15] Fleseriu M Hashim IA Karavitaki N Melmed S Murad MH Salvatori R. Hormonal Replacement in Hypopituitarism in Adults: An Endocrine Society Clinical Practice Guideline. J Clin Endocrinol Metab (2016) 101(11):3888–921. doi: 10.1210/jc.2016-2118 27736313

[16] Ma W Mao J Nie M Wang X Zheng J Liu Z. Gonadotropin Therapy Once a Week for Spermatogenesis in Hypogonadotropic Hypogonadism. Endocr Pract (2021) 27(11):1119–27. doi: 10.1016/j.eprac.2021.04.009 33915281

[17] Bidlingmaier M Friedrich N Emeny RT Spranger J Wolthers OD Roswall J. Reference Intervals for Insulin-Like Growth Factor-1 (Igf-I) From Birth to Senescence: Results From a Multicenter Study Using a New Automated Chemiluminescence IGF-I Immunoassay Conforming to Recent International Recommendations. J Clin Endocrinol Metab (2014) 99(5):1712–21. doi: 10.1210/jc.2013-3059 24606072

[18] Cooper TG Noonan E Von Eckardstein S Auger J Baker HW Behre HM. World Health Organization Reference Values for Human Semen Characteristics. Hum Reprod Update (2010) 16(3):231–45. doi: 10.1093/humupd/dmp048 19934213

[19] Cannarella R Crafa A La Vignera S Condorelli RA Calogero AE. Role of the GH-IGF1 Axis on the Hypothalamus-Pituitary-Testicular Axis Function: Lessons From Laron Syndrome. Endocr Connect (2021) 10(9):1006–17. doi: 10.1530/EC-21-0252 PMC8428041 34319907

[20] Tenuta M Carlomagno F Cangiano B Kanakis G Pozza C Sbardella E. Somatotropic-Testicular Axis: A Crosstalk Between GH/IGF-I and Gonadal Hormones During Development, Transition, and Adult Age. Andrology (2021) 9(1):168–84. doi: 10.1111/andr.12918 33021069

[21] Macdonald AA Herbison GP Showell M Farquhar CM. The Impact of Body Mass Index on Semen Parameters and Reproductive Hormones in Human Males: A Systematic Review With Meta-Analysis. Hum Reprod Update (2010) 16(3):293–311. doi: 10.1093/humupd/dmp047 19889752

[22] Rastrelli G Corona G Mannucci E Maggi M. Factors Affecting Spermatogenesis Upon Gonadotropin-Replacement Therapy: A Meta-Analytic Study. Andrology (2014) 2(6):794–808. doi: 10.1111/andr.262 25271205

[23] Wen J Jiangfeng M Min N Xi W Shuying L Bingqing Y. Desmopressin Suppresses Gonadotropin-Induced Spermatogenesis in Patients With Pituitary Stalk Interruption Syndrome: A Retrospective, Single-Center Cohort Study. Endocr Pract (2021) 27(2):124–30. doi: 10.1016/j.eprac.2020.08.001 33563411

[24] Liu Z Mao J Wu X Xu H Wang X Huang B. Efficacy and Outcome Predictors of Gonadotropin Treatment for Male Congenital Hypogonadotropic Hypogonadism: A Retrospective Study of 223 Patients. Med (Baltimore) (2016) 95(9):e2867. doi: 10.1097/MD.0000000000002867 PMC4782854 26945370

[25] Kulin HE Samojlik E Santen R Santner S. The Effect of Growth Hormone on the Leydig Cell Response to Chorionic Gonadotrophin in Boys With Hypopituitarism. Clin Endocrinol (Oxf) (1981) 15(5):463–72. doi: 10.1111/j.1365-2265.1981.tb00689.x 7326847

[26] Balducci R Toscano V Mangiantini A Bianchi P Guglielmi R Boscherini B. The Effect of Growth Hormone Administration on Testicular Response During Gonadotropin Therapy in Subjects With Combined Gonadotropin and Growth Hormone Deficiencies. Acta Endocrinol (Copenh) (1993) 128(1):19–23. doi: 10.1530/acta.0.1280019 8447190

